# Statins are related to impaired exercise capacity in males but not females

**DOI:** 10.1371/journal.pone.0179534

**Published:** 2017-06-15

**Authors:** Martin Bahls, Stefan Groß, Till Ittermann, Raila Busch, Sven Gläser, Ralf Ewert, Henry Völzke, Stephan B. Felix, Marcus Dörr

**Affiliations:** 1 University Medicine Greifswald, Department of Internal Medicine B, Greifswald, Germany; 2 DZHK (German Centre for Cardiovascular Research), Partner Site Greifswald, Greifswald, Germany; 3 University of Greifswald, Institute for Community Medicine, Greifswald, Germany; University of British Columbia, CANADA

## Abstract

**Background:**

Exercise and statins reduce cardiovascular disease (CVD). Exercise capacity may be assessed using cardiopulmonary exercise testing (CPET). Whether statin medication is associated with CPET parameters is unclear. We investigated if statins are related with exercise capacity during CPET in the general population.

**Methods:**

Cross-sectional data of two independent cohorts of the Study of Health in Pomerania (SHIP) were merged (n = 3,500; 50% males). Oxygen consumption (VO_2_) at peak exercise (VO_2_peak) and anaerobic threshold (VO_2_@AT) was assessed during symptom-limited CPET. Two linear regression models related VO_2_peak with statin usage were calculated. Model 1 adjusted for age, sex, previous myocardial infarction, and physical inactivity and model 2 additionally for body mass index, smoking, hypertension, diabetes and estimated glomerular filtration rate. Propensity score matching was used for validation.

**Results:**

Statin usage was associated with lower VO_2_peak (no statin: 2336; 95%-confidence interval [CI]: 2287–2,385 vs. statin 2090; 95%-CI: 2,031–2149 ml/min; P < .0001) and VO_2_@AT (no statin: 1,172; 95%-CI: 1,142–1,202 vs. statin: 1,111; 95%-CI: 1,075–1,147 ml/min; P = .0061) in males but not females (VO_2_peak: no statin: 1,467; 95%-CI: 1,417–1,517 vs. statin: 1,503; 95%-CI: 1,426–1,579 ml/min; P = 1.00 and VO_2_@AT: no statin: 854; 95%-CI: 824–885 vs. statin 864; 95%-CI: 817–911 ml/min; P = 1.00). Model 2 revealed similar results. Propensity scores analysis confirmed the results.

**Conclusion:**

In the general population present statin medication was related with impaired exercise capacity in males but not females. Sex specific effects of statins on cardiopulmonary exercise capacity deserve further research.

## Introduction

Cardiovascular diseases (CVD) are the most common cause of morbidity and mortality in Western societies [1]. Primary and secondary prevention strategies target predisposing risk factors (e.g. increased low-density lipoprotein [LDL] plasma concentrations or physical inactivity) using pharmacological and lifestyle-modification strategies. A prime example for pharmacological strategies is statin medication which is predominantly used to reduce LDL levels [2]. Possible non-pharmacological prevention modalities include increasing physical activity and dietary counselling. In the past a plethora of research has investigated the effectiveness and specificity of these prevention options [3,4]. However, possible interactions between drugs and lifestyle interventions are only beginning to be recognized [5].

Exercise is well known to increase endothelial nitric oxide bioavailability and improve endothelial function as well as decrease the overall inflammatory burden and vascular resistance [6–8]. The non-lipid pleiotropic effects of statins exert similar effects on these parameters [2,9]. Additionally, a previous randomized clinical trial reported that statin medication impaired training induced increases in exercise capacity [10]. Therefore, exercise and statins may interact with one another. Standardized cardiopulmonary exercise testing (CPET) is a standard method to determine exercise capacity. Currently there is a knowledge gap in the literature whether statins are related with CPET results. We explored the association of statin medication with CPET parameters in the general population. The aim of this study was to investigate if statins are associated with cardiorespiratory exercise capacity using cross-sectional data from two independent population-based cohorts (Study of Health in Pomerania [SHIP]). Based on the previous findings we hypothesized an inverse association between exercise capacity and statin medication.

## Methods

### Study design and participants

For this analysis data derived from two independent cohorts of SHIP in West Pomerania in the Northeast of Germany [11] were merged. Recruitment strategy and study design have been reported elsewhere [12]. Briefly, between October 1997 and May 2001 6,265 subjects (age 20 to 79) were invited to participate in the study (SHIP-0). At baseline a total of 4,308 men and women agreed to an in depth health screening (response 68.8%), which has previously been described [12]. This investigation included data from the first follow-up examination between March 2003 and July 2006 (SHIP-1). Ten years after SHIP-0 a second, independent cross-sectional population-based study was initiated in the same geographical region: SHIP-Trend-0. A stratified random sample of 8,016 adults between the ages of 20–79 years was drawn. Both studies were approved by the ethics committee of the University of Greifswald and comply with the Declaration of Helsinki. All participants gave written consent. SHIP data are publically available for scientific and quality control purposes. Data usage can be applied for via www.community-medicine.de.

Initially all subjects who choose to participate in CPET testing were included. After exclusion of subjects with missing data (n = 18), severe renal disease (estimated glomerular filtration rate [eGFR] < 30 ml/min) (n = 4), chronic lung disease (n = 68), bronchial asthma (n = 48), and chronic bronchitis (n = 122) a total of 3,500 subjects remained in the sample. Of these 1,752 were male (253 on statin medication) and 1,748 female (171 on statin medication).

### Interview, medical and laboratory examination

All assessments were done according to standardized protocols. Study nurses were certified semi-annually. Trained and certified staff used standardized computer-assisted interviews to ask the subjects about their age, sex, smoking habits, and physical inactivity. Smoking habits were classified either as current smoker or non-smoker. Physical inactivity was defined as being physically active for less than one hour per week.

Medical history of the subjects (including previous myocardial infarction) was assessed using a computer-assisted interview questionnaire. Diabetes was defined based on the self-reported use of antidiabetic medication (anatomic, therapeutic, and chemical [ATC] code: A10) in the last 7 days or a glycosylated hemoglobin level > 6.5%. Blood pressure was assessed after a 5 min resting period in sitting position. Systolic and diastolic blood pressure measurements were taken three times, with three minutes rest in between, on the right arm using a digital blood pressure monitor (HEM-705CP, Omron Corporation, Tokyo, Japan). The second and third reading were averaged and used as blood pressure for the specific subject. Hypertensive patients were identified by either self-reported antihypertensive medication (antihypertensive medication [ATC C02], diuretics [ATC C03], vasodilators [ATC C04], beta blockers [ATC C07], calcium channel blockers [ATC C08], angiotensin-converting enzyme blockers [ATC C09A]) or blood pressure measurement (systolic blood pressure > 140 mmHg or diastolic blood pressure > 90 mmHg). Body mass index (BMI) was calculated as body weight (kg) / height (m)^2^. Statin (ATC C10AA) medication was assessed via questionnaire and scanning of a unique drug identifier (pharmaceutical central number, Pharmazentralnummer, PZN). Non-fasting blood samples were taken and serum levels of cholesterol (chol), and LDL cholesterol were assessed photometrically (Hitachi 704, Roche, Mannheim, Germany). The estimated glomerular filtration rate (eGFR) was calculated according to Stevens et al. [13]: eGFR = 186 x (plasma creatinine concentration x 0.0113118)^-1.154^ x age^-0.203^; multiplied by 0.742 for female subjects [mL/min/1.73 m^2^].

### Exercise testing

Exercise capacity was assessed with a cycle ergometer (Ergoselect 100, Ergoline, Bitz, Germany) using a modified Jones protocol [14]. Briefly, 3 min of rest were followed by 1 min of unloaded cycling (20 Watts) at ~60 rpm. Thereafter, the workload was increased by 16 W every minute. Even though, subjects were encouraged to reach maximal exhaustion before, no encouragement was provided during the test. Exercise testing was stopped by the subject due to exhaustion or by the attending physician due to chest pain and/or ECG abnormalities. Twelve lead ECG was recorded during rest and every minute thereafter. Peak heart rate was determined based on the highest 10 s average during late exercise or early recovery.

### Gas exchange variables

During exercise testing tidal volume (V_E_), oxygen consumption (VO_2_), and carbon dioxide consumption (VCO_2_) were assessed on a breath-by-breath basis using an Oxycon Pro with a Rudolf’s mask (JÄGER/VIASYS Healthcare System, Hoechberg, Germany) and averaged over 10 s intervals. Pulse oximetry was monitored continuously. Maximal oxygen consumption (VO_2_peak) was defined as the highest 10 s average of VO_2_ during late exercise or early recovery. Oxygen consumption at the aerobic threshold (VO_2_@AT) was determined by the V-slope method as described previously [15]. Peak oxygen pulse (O_2_HRmax) was calculated as VO_2_peak divided by peak heart rate.

### Statistics

For sample baseline characteristics continuous data are expressed as median and 25^th^/75^th^ quantile. Nominal data are expressed as frequency and percentage. A directed acyclic graph was used to determine potential confounding and appropriately adjust for age, sex, previous myocardial infarction, physical inactivity and a potential statin*sex interaction (S1 Fig) [16,17]. In addition, a clinical model which adjusted for age, sex, previous MI, physical inactivity, eGFR, BMI, smoking, hypertension and diabetes was calculated. All associations with O_2_HR_max_ were further adjusted for beta blocker usage. The statin sex interaction term in the linear regression model was significant (p < .0001). Therefore, further analyses were stratified by sex. The Wilcoxon-Mann-Whitney-Test was used for descriptive continuous variables and Chi-square for nominal variables. Sex specific linear regression model were used to assess the association between statin usage and VO_2_peak, VO_2_@AT and O_2_HR_max_. Normality of residuals and homoscedasticity was visually checked using histograms, Q-Q plots, and residuals-vs-fitted plots. In addition, a quantile regression analysis in 5 percentile steps from the 5^th^ to the 95^th^ quantile was calculated. Further, as a sensitivity analysis a propensity score analysis was performed. Propensity scores based on sex, age, BMI, diabetes, hypertension, and previous myocardial infarction were calculated using a logistic regression [18]. A stringent caliper of 0.1 was used for a 1:1 matching of subjects taking statins and those who do not take statins. Thereafter, a two-sided paired t-test was used to compare VO_2_peak between statin-taking and non-taking subjects. All calculations were done in SAS 9.4 (SAS Institute, Cary, NC, USA) or STATA13.1 (Stata Corporation, College Station, TX, USA). All results are given as least-square means and 95%-confidence interval (CI) unless otherwise indicated. Bonferroni adjustment for multiple testing was used when appropriate for group comparisons. Significance was determined by P < .05.

## Results

### General characteristics

Descriptive statics of SHIP-1 and SHIP-T are provided in S1 Table and S2 Table. Of the 3,500 analyzed subjects in the merged data set a total of 1,752 were male (253 [14.4%] on statin medication) and 1,748 female (171 [9.8%] on statin medication) (Table 1). Study participants taking statins were significantly older, smoked less, and had higher BMI, more often hypertension as well as diabetes mellitus. Further, median plasma LDL and cholesterol concentrations were lower, while median TG concentration was higher in statin compared to non-statin taking subjects. Values for VO_2_peak, VO_2_@AT and eGFR were lower in statins users. Also, subjects on statins were more likely to receive beta blockers, calcium-channel blockers and angiotensin-converting enzyme blockers. Interestingly, in males but not in females median diastolic blood pressure was lower in statin users compared to non-users. However, females on statins had significantly higher median systolic blood pressure compared to females without statins. No such difference was found in males. Median O_2_HR_max_ was significantly lower in males with statins compared to male subjects without this drug, while no difference was seen for females.

**Table 1:** Descriptive statistics.

	Males (n = 1,752)			Females (n = 1,748)		
	Statin users (n = 253)	Non-users (n = 1,499)	P	Statin users (n = 171)	Non-users (n = 1,577)	P
Age (years)	66 (57; 72)	50 (40; 61)	< .0001	65 (59; 71)	50 (40; 60)	< .0001
BMI (kg/m2)	29.38 (26.97; 31.78)	27.80 (25.30; 30.60)	< .0001	28.93 (26.29; 32.67)	25.96 (23.08; 29.76)	< .0001
Hypertension (%)	87.7	49.4	< .0001	74.3	33.3	< .0001
Myocardial infarction (%)	21.3	1.4	< .0001	4.1	0.4	< .0001
Diabetes mellitus (%)	29.6	7.1	< .0001	22.8	4.9	< .0001
**Diastolic BP (mmHg)**	**77 (71; 84)**	**81 (75; 88)**	**< .0001**	**76 (70; 81)**	**76 (70; 82)**	**0.6744**
**Systolic BP (mmHg)**	**134 (124; 144)**	**133 (123; 144)**	**0.6503**	**127 (117; 139)**	**120 (110; 132)**	**< .0001**
LDL (mmol/l)	2.64 (2.18; 3.19)	3.58 (2.98; 4.18)	< .0001	2.97 (2.55; 3.53)	3.40 (2.79; 4.12)	< .0001
TG (mmol/l)	1.72 (1.21; 2.54)	1.50 (1.01; 2.30)	0.002	1.47 (1.11; 1.96)	1.18 (0.84; 1.68)	< .0001
Chol (mmol/l)	4.50 (3.90; 5.20)	5.60 (4.80; 6.30)	< .0001	5.20 (4.60; 5.80)	5.60 (4.90; 6.31)	< .0001
Smoking (%)	14.2	25.2		9.9	22.7	
VO_2_max (ml/min)	1922 (1573; 2237)	2450 (2062; 2868)	< .0001	1400 (1159; 1600)	1600 (1366; 1870)	< .0001
VO_2_@AT (ml/min)	1050 (850; 1200)	1200 (1000; 1400)	< .0001	800 (700; 950)	900 (800; 1000)	< .0001
**O**_**2**_**HRmax (ml/beat)**	**14.6 (12.8; 16.7)**	**15.7 (13.8; 17.9)**	**< .0001**	**10.7 (9.5; 12.2)**	**10.5 (9.3; 12.0)**	**0.2635**
Beta Blocker (%)	60.1	16.3	< .0001	53.8	18.7	< .0001
Calcium Channel Blockers (%)	17	3.3	< .0001	7.6	2.4	< .0001
Angiotensin-converting enzyme blockers (%)	47	10.5	< .0001	25.7	7.2	< .0001
Physical inactivity (%)	41.9	38.4	0.2952	32.7	35.38	0.4932
eGFR	79.5 (67.4; 90.7)	89.46 (78.5; 101.8)	< .0001	75.1 (64.1; 85.6)	85.2 (74.1; 99.6)	< .0001

### The association between statin medication and VO_2_peak

Since a significant sex–statin interaction was observed in the initial linear regression model all further group comparisons were stratified by sex (p < .001) (S3 Table). Results for the individual studies (SHIP-1 and SHIP-T) are provided in S4 and S5 Tables. In model 1 statin medication was inversely associated with VO_2_peak in males (no statin: 2,337; 95%-confidence interval [CI]: 2,287–2,386 vs. statin: 2,091; 95%-CI: 2,032–2,150 ml/min; P < .0001) but not females (no statin: 1,467; 95%-CI: 1,417–1,517 vs. statin: 1,503: 95%-CI: 1,426–1,579 ml/min; P = 1.00). Model 2 showed similar results (male no statin: 2,268; 95%-CI: 2,214–2,321 vs. male statin: 2,035; 95%-CI: 1,975–2,096 ml/min; P < .0001; female no statin: 1,388; 95%-CI: 1,334–1,443 vs. female statin: 1,434; 95%-CI: 1,356–1,511 ml/min; P = 1.00).

**Fig 1:**
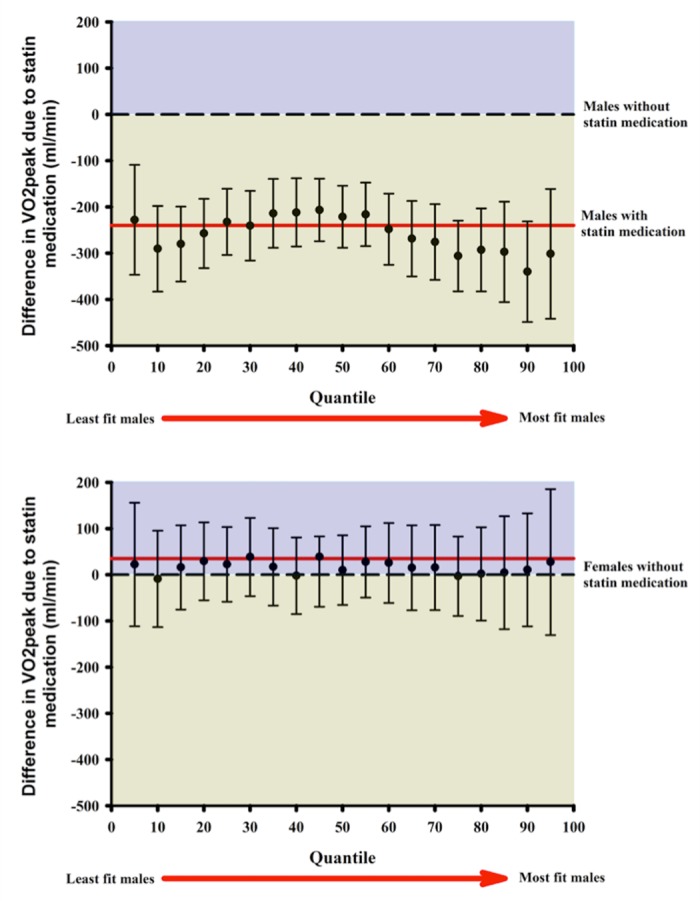
**Association between statin medication with VO**_**2**_**peak for males (A) and females (B).** The dashed line indicates a β-coefficient of “0”, which means that there are no significant differences in VO_2_peak between statin users and non-users. The red line is representative of the overall β (i.e. the observed difference in VO_2_peak between statin users and non-users). The quantiles on the x-axis are representative of exercise capacity. Subjects with the lowest VO_2_peak are on the left and while the one with highest exercise capacity are on the right. Statin medication was associated with a significantly reduced VO_2_peak in male between the 5^th^ and 95^th^ quantiles. For females no significant differences were observed.

Quantile regression analysis revealed a consistent effect throughout the range of observed values for both sexes. Specifically, statin usage was associated with a significantly reduced VO_2_peak for all observed values between the 5^th^ and the 95^th^ percentile in males. Quantile regression analysis also confirmed that statin usage was not associated with reduced VO_2_peak in females for the range of observed values (Fig 1).

### The association between statin medication and VO_2_@AT

Statin medication was associated with reduced VO_2_@AT in males (no statin: 1,172; 95%-CI: 1,142–1,202 vs. statin: 1,111; 95%-CI: 1,075–1,147 ml/min; P = .0061) but not females (no statin: 854; 95%-CI: 824–885 vs. statin 864; 95%-CI: 817–911 ml/min; P = 1.00). In Model 2 a significantly lower VO_2_@AT was seen in statin taking males (no statin: 1,153; 95%-CI: 1,120–1,186 vs. statin: 1,089; 95%-CI: 1,051–1,126 ml/min; P = .0032) but not females (no statin: 835; 95%-CI: 801–868 vs. statin: 840; 95%-CI: 792–888 ml/min; P = 1.00).

### Interaction between physical inactivity with statin medication

In order to investigate whether differences in physical activity may be responsible for our results we explored the three-way interaction between physical inactivity, statins, and sex. This interaction revealed to be significant in model 1 (P < .0001) and model 2 (P < .0001). Thus, a post-hoc analysis between groups adjusted for multiple testing was conducted. In model 1 physically active non-statin taking males (inactive: 2,236 95%-CI: 2,180–2,291 vs. active: 2,440 95%-CI: 2,389–2,493 ml/min; P < .0001) and females (inactive: 1,407 95%-CI: 1,351–1,464 vs. active: 1,549 95%-CI: 1,497–1,601 ml/min; P < .0001) had significantly higher VO_2_peak compared to their inactive counterparts. In statin taking males (inactive: 2,061 95%-CI: 1,977–2,146 ml/min vs. active: 2,171 95%-CI: 2,098–2,245 ml/min; P = 0.9776) and females (inactive: 1,528 95%-CI: 1,412–1,644 ml/min vs. active: 1,563 95%-CI: 1452–1627 ml/min; P = 1.0) no significant difference was observed due to physical activity. The results for model 2 were similar. Specifically, physically active males (inactive– 2,171 95%-CI: 2,112–2,230 ml/min vs. active– 2,367 95%-CI: 2,311–2,424 ml/min; P < .0001) and females (inactive– 1,345 95%-CI: 1,285–1,405 ml/min vs. active– 1,468 95%-CI: 1,410–1,525 ml/min; < .0001) had significantly higher VO_2_peak compared to inactive individuals. For statin taking males (inactive– 2,015 95%-CI: 1,930–2,100 ml/min vs. active– 2,102 95%CI: 2,028–2,176 ml/min; P = 1.00) and females (inactive– 1,465 95%CI: 1,350–1,580 ml/min vs. active– 1,489 95%-CI: 1,399–1,579 ml/min; P = 1.00) physically activity had no effect on VO_2_peak.

### The association between statin medication and O_2_HR_max_

In model 1 statin medication was associated with a significantly reduced O_2_HR_max_ in males (no statin: 15.5; 95%-CI: 15.2–15.8 vs. statin: 14.6; 95%-CI: 14.2–15.0 ml/beat; P < .0001) but not females (no statin: 10.3; 95%-CI: 9.9–10.6 vs. statin: 10.5; 95%-CI: 10.0–11.0 ml/beat; P = 1.00). Model 2 also showed that for males (no statin: 15.5; 95%-CI: 15.2–15.9 vs. statin: 14.5; 95%-CI: 14.1–14.9 ml/beat; P < .0001) but not females (no statin: 10.3; 95%-CI: 9.9–10.6 vs. statin: 10.5; 95%-CI: 10.0–11.0 ml/beat; P = 1.00) statin usage was inversely associated with O_2_HR_max_.

### The association between cardiopulmonary exercise capacity and lipids

Circulating LDL and cholesterol concentration was inversely associated with VO_2_peak in men (LDL: -33.51 95%-CI: -59.26 –-7.75 ml/mmol/l; P = 0.0108 and cholesterol: -27.29 95%-CI: -48.64–-5.93 ml/mmol/l; P = 0.0123) but not women (LDL: -14.65 95%-CI: -31.65–2.35; P = 0.0911 and cholesterol: 14.76 95%-CI: -29-8–0.25; P = 0.054).

### Sensitivity analysis using propensity scores

In our sample a total of 253 male subjects took statins. A total of 229 individuals were matched according to their likelihood to receive a statin. The discrepancy between the 253 males in the cohort and 232 matched is likely due to the stringent matching caliper of 0.1. According to this approach, male subjects on statin medication had significantly lower VO_2_peak compared to subjects without this medication by 110.5 ml/min (95%-CI: 31.0–111.0 ml/min; P < .001). All 171 female subjects taking statins could be matched to controls. No significant difference between groups was found (mean difference 28.4; 95%-CI: -87.6–30.8 ml/min; P = .34). Therefore, the propensity score analysis confirmed our findings.

## Discussion

Statins and exercise are life-saving therapeutics with systemic pleiotropic effects. This investigation explored a potential association between statins and cardiopulmonary exercise testing capacity in two independent cross-sectional population-based cohorts from Northeast Germany. We report that statin usage was inversely related with VO_2_peak and VO_2_@AT in males but not females. Male statin users had significantly lower VO_2_peak between the 5^th^ and 95^th^ percentile. In females no association of statins with VO_2_peak and VO_2_@AT was observed throughout the range of observed values. In line with these findings, statin medication was also associated with reduced oxygen pulse in males but not females. In addition, the results of the investigated three-way interaction between sex, physical inactivity, and statin medication indicate that the relation between statins and reduced VO_2_peak was dependent on sex but independent of physical activity. This is in contrast to previous findings which reported that physical activity decreased in elderly men after initiation of statin treatment [19].

Maximal oxygen consumption is a very well established predictor for mortality in healthy populations [20] and in patients with coronary heart disease [21]. The positive outcomes of participating in structured exercise programs for patients who experienced acute cardiovascular events are well established [22,23]. Also, patients with heart failure [24] and coronary heart disease [25], who receive statins, cannot just safely participate in endurance and interval training protocols, but also increase their cardiopulmonary exercise capacity. However, some previous studies suggest that statins may also worsen cardiac function. In a very small trial, 14 patients without previous myocardial function and heart failure were given 20 mg of atorvastatin per day [26]. A total of 10 patients showed a worsening of diastolic ventricular function (e.g. 10% decrease in the E/A ratio, a 10% increase in E-wave DT, or a 10% increase in IVRT). The authors of the trial reported that affected patients had very low coenzyme Q_10_ which can be further reduced by statins [27]. In line with this, a very recent clinical trial with 420 heart failure patients reported that coenzyme Q_10_ supplementation significantly reduced major adverse cardiovascular events [28].

Statins are also recommended for subjects without cardiovascular disease but who have an increased risk for CVD [29,30]. The separate efficacy of either exercise or statins in primary CVD prevention has been well established [31,32]. To demonstrate the suitability of statins for subjects without cardiovascular disease, previous studies have reported that statins did not decrease VO_2_peak [33]. However, these results have to be taken with caution since they are based on a small sample size of 28. Also, large epidemiological studies reported an additive effect of statins and physical activity on mortality reduction in dyslipidaemic and hypertensive individuals [34,35]. Interestingly, Kokkinos et al. reported that the fittest study participants had the lowest risk of mortality independent of statin usage [34]. However, while in secondary disease prevention, subjects were able to increase their VO_2_peak with exercise training; this may not be the case in primary prevention. Mikus et al. recently showed that statins impaired the beneficial effects of a twelve week supervised aerobic endurance exercise training protocol in obese subjects [10]. Their investigation was the first to compare two exercise groups to assess the additional effect of statins on top of exercise on trainability. Statins inhibited an increase in VO_2_peak. We report that in males from a general population cohort current statin usage was associated with significantly lower VO_2_peak independent of age, previous myocardial infarction, physical activity behavior, BMI, diabetes, hypertension, beta blocker usage, calcium channel blockers, and angiotensin converting enzyme blockers. Further research is warranted to understand which subjects benefit most from statins and/or exercise.

In addition to lower VO_2_peak, statin usage was related with lower VO_2_@AT in males, but not females. In contrast to our data, a human trial which investigated cardiopulmonary exercise capacity in patients with statin induced myotoxicity reported that the aerobic threshold was not impacted by statin medication in their control group [36]. The authors speculated that an increased resting respiratory exchange ratio due to statins may impair the lipid metabolism during exercise. Our findings point to a more nuanced interpretation, since our results suggest sex specific relation of statins with cardiopulmonary exercise capacity. Nonetheless, we acknowledge that our results are based on observational data and not based on a randomized controlled trial (RCT). Hence, our results are subject to bias and confounding. Overall, these effects deserve further research.

Several studies have explored the impact of statins on physical fitness and activity [10,19,34,37–44]. However, these investigations are very heterogeneous in terms of participants and experimental design. For example, Sinzinger and O’Grady examined the impact of statins on muscle pain in elite athletes with familial hypercholesterolemia. They reported that merely 20% tolerated statin treatment without reporting any adverse effects [37]. Contrarily, in healthy individuals with increased LDL concentration, simvastatin treatment for 12 weeks was not associated with decreases in cardiopulmonary exercise capacity and muscle function [38]. In a large observational study with 1,201 patients undergoing cardiac rehabilitation significant improvements in VO_2_peak were reported for statin users and non-users alike [40]. In a large very recent epidemiological study Williams and Thompson investigated the role of statins in more than 60,000 runners and walkers [44]. Statins did not influence exercise activity or duration in individuals who developed hypercholesterolemia. Lastly, in the The Henry Ford Exercise Testing (FIT) Project statin usage was not associated with decreased VO_2_peak in males or females [42]. These data suggest that statins may not adversely influence physical activity and capacity.

The sex specific results of our analysis are of particular interest. Even though, we acknowledge that our population-based approach is observational, it is well established that statin therapy increases the risk for skeletal muscle myopathy. This side effect primarily impacts glycolytic type II muscle and not oxidative type I fibers [45,46]. Since women have more type I fibers compared to men [47], males may be at a higher risk to experience statin induced muscle damage. Interestingly, previous experimental evidence demonstrated higher rates of statin induced muscle problems in women [48]. A second possible explanation is that lipophilic statins (i.e. lovastatin, simvastatin, fluvastatin, atorvastatin, and pivastatin) are metabolized in first-pass in the liver through the hepatic cytochrome enzyme system [49]. The enzyme responsible for this reaction is cytochrome P450 3A4 (CYP3A4). Women have been found to have higher concentrations of CYP3A4 [50,51]. Hence, females may be able to metabolize statins more quickly and thus reduce their efficacy. The third possible reason as to why a sex-specific association was observed in our investigation may be due to the difference in O_2_HR_max_. While statin taking males had lower O_2_HR_max_ compared to non-users, female statin takers and non-takers had similar O_2_HR_max_. Since O_2_HR_max_ is strongly related with left ventricular ejection fraction (LVEF), the inverse association between statin usage and VO_2_peak in males may be a result of lower LVEF which was not evident in female statin users. A reduced LVEF in male statin users may be partly responsible for their lower cardiopulmonary exercise capacity. Overall, at least three explanations (e.g. effect of statins on type I skeletal muscle fibers, sex-specific CYP3A4 concentration and/or differences in O_2_HR_max_) for the observed sex-specific associations between statins and CPET parameters are likely. Nonetheless, we are currently unable to unequivocally determine the underlying reason for our observations.

Our findings are in contrast to previous observations that reported an increased incidence of statin adverse effects in women compared to men [48]. Further, in a randomized controlled trial (RCT) women receiving statins experienced more fatigue compared to men [52]. These observed side effects of statins were related to alterations in mitochondrial function [48]. Since mitochondrial function is associated with cardiopulmonary exercise capacity [53], one would propose that statins show an inverse association with VO_2_peak. One may speculate the different results are due to the fact that we analyzed data from a general populated based cohort while the above mentioned RCT recruited subjects from a primary prevention population without overt cardiovascular disease or diabetes.

One of the reasons for the statin treatment in our population was most likely increased circulating LDL and cholesterol. Altered lipid metabolism is an early hallmark of subclinical cardiovascular disease and may therefore influence cardiopulmonary exercise capacity. We assessed the association between circulating LDL and cholesterol with VO_2_peak in subjects not taking statin medication. We report an inverse association between VO_2_peak to LDL and cholesterol in males but not females. Interestingly, the parameter estimate for LDL points to an inverse association for both sexes. This agrees with the common thought that increased LDL is associated with subclinical CVD and therefore reduced cardiopulmonary exercise capacity. However, the parameter estimate for the association between VO_2_peak and cholesterol is inverse for males but positive for females. Future analyses need to verify this finding as it may just be a result of chance.

We acknowledge that based on the cross-sectional analysis of observational data we cannot conclusively determine the direction of the investigated associations. Further, the cross sectional design only allows for the comparison of current statin users and non-users. Since CPET was voluntary in our cohort, a potential bias may have been introduced due to more fit individuals selecting to participate in this test. Nonetheless, the strength of our study is the population-based design. This may allow drawing inferences for the general population from our results. However, we are aware that only a small number of participants took statins, which in turn may be a realistic assumption about the general population. In addition, we employed a highly standardized quality control during the course of the study. Lastly, we recognize our analysis would benefit from information regarding the duration and dose of statin treatment as well as possible muscle symptoms. Unfortunately this data is not available for our cohorts. Our findings may be influenced by residual confounding since we included variables which are strongly predictive of cardiopulmonary exercise capacity and have a large degree of variability. Most importantly we were unable to adjust to lipid levels prior to statin treatment.

One may argue that the observed associations between VO_2_peak and VO_2_@AT with present statin medication are the result of an observational bias due to comparing healthy with sick sub-populations. To investigate whether this was the case, we used propensity scores in a sensitivity analysis as a method to overcome this potential shortcoming. The propensity scores were calculated using a logistic regression adjusted for age, sex, BMI, diabetes, hypertension, previous myocardial infarction and usage of beta-blockers, calcium channel blockers, and angiotensin-converting enzyme blockers. Thereafter, we matched subjects with and without statins according to a very stringent caliper of 0.1. This means that the difference in propensity scores has to be less than 0.1. This method is more rigorous than using nearest neighbor matching which is exemplified by the fact that we were unable to match all statin taking subjects. A paired t-test between matched male subject pairs demonstrated that statin users had significantly lower VO_2_peak and VO_2_@AT compared to non-users. No significant difference was observed for females. Therefore, the propensity score analysis confirmed the results of the regression analysis.

## Conclusion

We report that present statin medication was related with impaired cardiopulmonary exercise capacity in male but not female subjects in a cross-sectional population based study from northeast Germany. Based on previous research, statins may inhibit increases in VO_2_peak due to exercise training in primary, but not secondary disease prevention. Therefore, whether subjects in primary cardiovascular prevention aiming to increase their cardiopulmonary exercise capacity should take this medication deserves further research. Future research should use a randomized design to test whether our observational results can be confirmed to assess whether exercise and not drug usage is more suitable as a first frontier in primary disease prevention.

## Supporting information

S1 Fig Directed acyclic graph to model the association between statin usage and cardiorespiratory exercise capacity. Variables in red are included into the model while variables in blue are considered mediators.
(JPG)

S1 Table Population description of SHIP-1. (PDF)

S2 Table Population description of SHIP-T. (PDF)

S3 Table Results of interaction analysis. (PDF)

S4 Table Association between statin usage and VO2peak SHIP-1. (PDF)

S5 Table Association between statin usage and VO2peak in SHIP-T. (PDF)
