# Supplementary figures and images for: Statins are related to impaired exercise capacity in males but not females
Source: PLoS One. 2017 Jun 15;12(6):e0179534. doi: 10.1371/journal.pone.0179534 (PMC5472298; doi:10.1371/journal.pone.0179534)

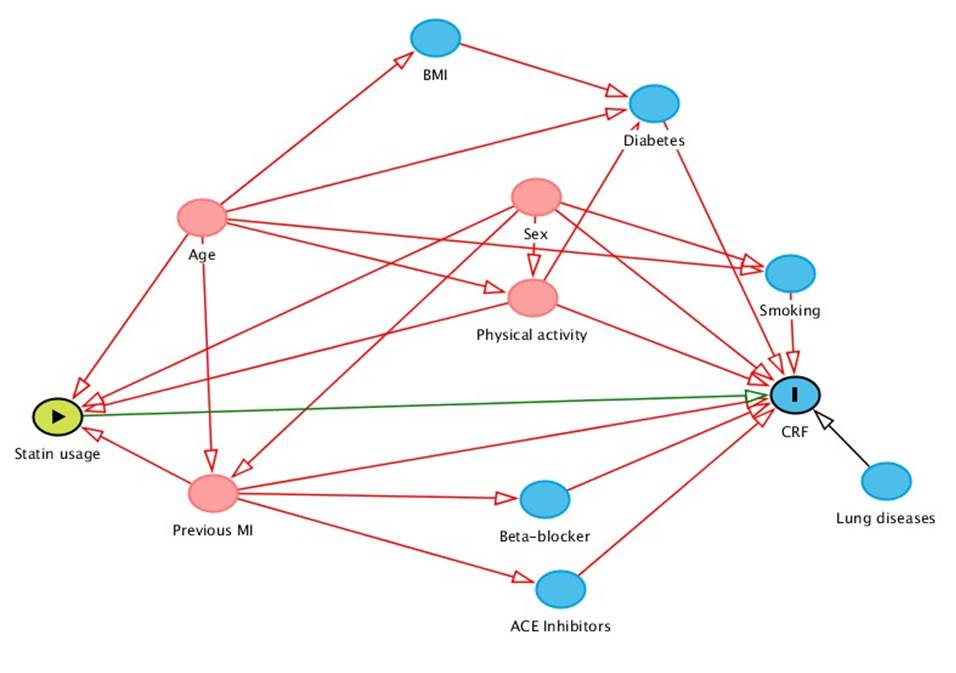

Supplement: S1 Fig — Variables in red are included into the model while variables in blue are considered mediators. (JPG) [file pone.0179534.s001.jpg]
