# Supplementary material for: Statins are related to impaired exercise capacity in males but not females
Source: PLoS One. 2017 Jun 15;12(6):e0179534. doi: 10.1371/journal.pone.0179534 (PMC5472298; doi:10.1371/journal.pone.0179534)
Supplement: S1 Table — (PDF) [file pone.0179534.s002.pdf]

S Table 1 – Population description of SHIP-1

	Males (n = 584)			Females (n = 603)		
	Statin users (n = 80)	Non-users (n = 504)	P	Statin users (n = 58)	Non-users (n = 545)	P
Age (years)	66 (55; 71)	51 (39; 61)	<.0001	64 (60; 70)	51 (40; 60)	<.0001
BMI (kg/m2)	28.8 (26.1; 30.8)	27.5 (25.1; 30.6)	0.1191	29.6 (26.4; 33.7)	25.8 (23.0; 29.6)	<.0001
Hypertension (%)	82.5	52.4	<.0001	70.7	36.9	<.0001
Myocardial infarction (%)	22.5	2.0	<.0001	3.5	0.6	<.0001
Diabetes mellitus (%)	17.5	6.7	<.0001	20.7	3.1	<.0001
**Diastolic BP (mmHg)**	**80 (72; 86)**	**85 (78; 91)**	**<.0001**	**77 (73; 85)**	**80 (74; 86)**	**0.2372**
**Systolic BP (mmHg)**	**130 (121; 140)**	**134 (124; 146)**	**0.0574**	**130 (121; 140)**	**124 (115; 137)**	**0.0018**
LDL (mmol/l)	2.8 (2.4; 3.3)	3.7 (3.1; 4.3)	<.0001	3.2 (2.6; 3.8)	3.5 (2.8; 4.3)	0.0354
TG (mmol/l)	1.7 (1.4; 2.5)	1.8 (1.2; 2.6)	0.6764	1.5 (1.1; 2.6)	1.2 (0.8; 1.8)	0.0006
Chol (mmol/l)	4.7 (4.0; 5.5)	5.7 (5.0; 6.4)	<.0001	5.5 (4.8; 6.0)	5.6 (4.9; 6.4)	0.1561
Smoking (%)	60.0	42.3	<.0001	15.5	17.8	<.0001
VO_2max (ml/min)	1893 (1590; 2245)	2424 (2017; 2813)	<.0001	1448 (1200; 1600)	1600 (1400; 1850)	<.0001
VO_2@AT (ml/min)	1150 (1000; 1300)	1250 (1100; 1500)	0.0003	900 (750; 1000)	950 (803; 1100)	0.0118
**O_2HRmax (ml/beat)**	**15.0 (13.3; 16.6)**	**15.6 (13.7; 18.0)**	**0.0317**	**11.3 (9.8; 13.0)**	**10.6 (9.6; 12.0)**	**0.1788**
Beta Blocker (%)	56.3	14.9	<.0001	46.6	18.5	<.0001
Angiotensin-converting enzyme blockers (%)	46.3	10.3	<.0001	15.5	7.3	<.0001
Physical inactivity (%)	61.3	57.9	0.5771	56.9	52.1	0.4883
eGFR	77 (68; 89)	88 (78; 99)	<.0001	71 (63; 83)	83 (72; 95)	<.0001
