# Supplementary material for: Statins are related to impaired exercise capacity in males but not females
Source: PLoS One. 2017 Jun 15;12(6):e0179534. doi: 10.1371/journal.pone.0179534 (PMC5472298; doi:10.1371/journal.pone.0179534)
Supplement: S2 Table — (PDF) [file pone.0179534.s003.pdf]

S2 Table – Population description of SHIP-T

	Males (n = 1,168)			Females (n = 1,145)		
	Statin users (n = 173)	Non-users (n = 995)	P	Statin users (n = 113)	Non-users (n = 1,032)	P
Age (years)	67 (58; 72)	50 (40; 61)	<.0001	66 (59; 71)	49 (40; 60)	<.0001
BMI (kg/m2)	29.5 (27.3; 32.2)	27.9 (25.4; 30.6)	<.0001	28.4 (26.2; 31.8)	26.0 (23.1; 29.9)	<.0001
Hypertension (%)	89.6	47.3	<.0001	75.2	31.4	<.0001
Myocardial infarction (%)	20.8	1.1	<.0001	3.5	0.4	<.0001
Diabetes mellitus (%)	35.3	7.3	<.0001	23.9	0.6	<.0001
**Diastolic BP (mmHg)**	**76 (70; 83)**	**80 (74; 87)**	**<.0001**	**74 (69; 80)**	**74(68; 80)**	**0.7421**
**Systolic BP (mmHg)**	**136 (124; 145)**	**132 (123; 143)**	**0.0645**	**125 (112; 138)**	**117 (108;130)**	**0.0001**
LDL (mmol/l)	2.56 (2.06; 3.14)	3.53 (2.92; 4.09)	<.0001	2.84 (2.53; 3.43)	3.37 (2.77; 4.06)	<.0001
TG (mmol/l)	1.68 (1.18; 2.55)	1.40 (0.96; 2.13)	0.0007	1.47 (1.11; 1.86)	1.17 (0.86; 1.64)	<.0001
Chol (mmol/l)	4.50 (3.80; 5.20)	5.50 (4.70; 6.20)	<.0001	5.10 (4.50; 5.70)	5.60 (4.90; 6.30)	<.0001
Smoking (%)	12.7	24.6	<.0001	9.7	22.4	<.0001
VO_2max (ml/min)	1950 (1562; 2200)	2458 (2088; 2890)	<.0001	1362 (1150; 1550)	1600 (1352; 1883)	<.0001
VO_2@AT (ml/min)	1000 (850; 200)	1200 (1000; 1300)	<.0001	800 (700; 900)	850 (750; 1000)	0.0013
**O_2HRmax (ml/beat)**	**14.4 (12.5; 16.8)**	**15.7 (13.8; 17.9)**	**<.0001**	**10.6 (9.3; 12.0)**	**10.5 (9.0; 12.0)**	**0.6786**
Beta Blocker (%)	61.8	17.0		57.5	18.8	<.0001
Angiotensin-converting enzyme blockers (%)	47.4	10.7	<.0001	31.0	7.1	<.0001
Physical inactivity (%)	32.9	28.5	0.2398	20.4	26,6	0.1539
eGFR	80.5 (67.3; 91.4)	90.2 (78.7; 103.2)	<.0001	77.8 (65.0; 86.7)	87.0 (75.5; 101.3)	<.0001
