# Supplementary material for: Statins are related to impaired exercise capacity in males but not females
Source: PLoS One. 2017 Jun 15;12(6):e0179534. doi: 10.1371/journal.pone.0179534 (PMC5472298; doi:10.1371/journal.pone.0179534)
Supplement: S3 Table — (PDF) [file pone.0179534.s004.pdf]

S3 Table – Results of interaction analysis

Model	Source	DF	Type III SS	Mean Square	F	P
Basic model	Statin usage	1	3382077.7	3382077.7	20.12	<.0001
	Previous myocardial infarction	1	3220258.2	3220258.2	19.16	<.0001
	Sex	1	184054594	184054594	1095.15	<.0001
	Age	1	209187926	209187926	1244.7	<.0001
	Physical inactivity	1	21355948.1	21355948.1	127.07	<.0001
	Statin sex interaction	1	6933478.4	6933478.4	41.26	<.0001
Clinical model	Statin usage	1	2576639.3	2576639.3	15.89	<.0001
	Previous myocardial infarction	1	3296619.1	3296619.1	20.33	<.0001
	Sex	1	187743060	187743060	1157.57	<.0001
	Age	1	189706666	189706666	1169.67	<.0001
	Physical inactivity	1	17217222.4	17217222.4	106.16	<.0001
	Smoking	1	20646357.3	20646357.3	127.3	<.0001
	Diabetes	1	584960.1	584960.1	3.61	0.0576
	Hypertension	1	250148.4	250148.4	1.54	0.2144
	Statin sex interaction	1	6759697.7	6759697.7	41.68	<.0001
