# Supplementary material for: Statins are related to impaired exercise capacity in males but not females
Source: PLoS One. 2017 Jun 15;12(6):e0179534. doi: 10.1371/journal.pone.0179534 (PMC5472298; doi:10.1371/journal.pone.0179534)
Supplement: S4 Table — (PDF) [file pone.0179534.s005.pdf]

S4 Table – Association between statin usage and VO_2peak SHIP-1

Model	Sex	Variable	DF	Parameter estimate	95%-CI		P
Basic model	Male (n = 584)	Intercept	1	4028	3838	4219	<.0001
		Statin	1	-163	-287	-39	.0100
		Previous myocardial infarction	1	-159	-351	32	.1033
		Age	1	-24	-27	-21	<.0001
		Physical inactivity	1	211	132	290	<.0001
	Female (n = 603)	Intercept	1	2404	2282	2526	<.0001
		Statin	1	-34	-117	49	.4179
		Previous myocardial infarction	1	-297	-556	-38	.0247
		Age	1	-12	-14	-10	<.0001
		Physical inactivity	1	100	53	146	<.0001
Clinical model	Male (n = 584)	Intercept	1	4134	3935	4334	<.0001
		Statin	1	-142	-264	-19	.0233
		Previous myocardial infarction	1	-159	-348	31	.1002
		Age	1	-25	-28	-21	<.0001
		Physical inactivity	1	190	112	268	<.0001
		Smoking	1	-117	-232	-65	<.0001
		Diabetes	1	-88	-232	56	.2325
		Hypertension	1	-41	-125	43	.3402
	Female (n = 603)	Intercept	1	2450	2319	2580	<.0001
		Statin	1	-47	-133	37	.2713
		Previous myocardial infarction	1	-291	-550	-32	.0278
		Age	1	-13	-15	-11	<.0001
		Physical inactivity	1	100	53	147	<.0001
		Smoking	1	-19	-49	11	.2105
		Diabetes	1	62	-51	175	.2846
		Hypertension	1	28	-25	81	.2969
