# Supplementary material for: Statins are related to impaired exercise capacity in males but not females
Source: PLoS One. 2017 Jun 15;12(6):e0179534. doi: 10.1371/journal.pone.0179534 (PMC5472298; doi:10.1371/journal.pone.0179534)
Supplement: S5 Table — (PDF) [file pone.0179534.s006.pdf]

S5 Table – Association between statin usage and VO_2peak in SHIP-T

Model	Sex	Variable	DF	Parameter estimate	95%-CI		P
Basic model	Male (n = 1168)	Intercept	1	3583	3469	3697	<.0001
		Statin	1	-167	-254	-80	.0002
		Previous myocardial infarction	1	-256	-406	-106	.0008
		Age	1	-24	-26	-22	<.0001
		Physical inactivity	1	185	125	246	<.0001
	Female (n = 1145)	Intercept	1	2169	2093	2246	<.0001
		Statin	1	-63	-130	3	0.0608
		Previous myocardial infarction	1	34	-180	247	.7572
		Age	1	-13	-15	-12	<.0001
		Physical inactivity	1	169	126	212	<.0001
Clinical model	Male (n = 1168)	Intercept	1	3173	3031	3315	<.0001
		Statin	1	-151	-238	-63	.0007
		Previous myocardial infarction	1	-261	-406	-116	.0004
		Age	1	-26	-28	-24	<.0001
		Physical inactivity	1	165	107	224	<.0001
		Smoking	1	-304	-238	-370	<.0001
		Diabetes	1	-39	-129	50	.3910
		Hypertension	1	-26	-87	35	.4078
	Female (n = 1145)	Intercept	1	2027	1927	2127	<.0001
		Statin	1	-55	-122	12	.1101
		Previous myocardial infarction	1	65	-148	277	.5516
		Age	1	-14	-15	-12	<.0001
		Physical inactivity	1	159	116	202	<.0001
		Smoking	1	-99	-146	-51	<.0001
		Diabetes	1	-15	-89	58	.6858
		Hypertension	1	-34	-79	10	.1315
